# Synthesis of Poly(3-vinylpyridine)-*Block*-Polystyrene Diblock Copolymers via Surfactant-Free RAFT Emulsion Polymerization

**DOI:** 10.3390/ma12193145

**Published:** 2019-09-26

**Authors:** Katharina Nieswandt, Prokopios Georgopanos, Clarissa Abetz, Volkan Filiz, Volker Abetz

**Affiliations:** 1 Helmholtz-Zentrum Geesthacht, Institute of Polymer Research, Max-Planck-Straße 1, 21502 Geesthacht, Germany; katharina.nieswandt@hzg.de (K.N.); clarissa.abetz@hzg.de (C.A.); volkan.filiz@hzg.de (V.F.); 2 Institute of Physical Chemistry, University of Hamburg, Martin-Luther-King-Platz 6, 20146 Hamburg, Germany

**Keywords:** RAFT polymerization, polymerization-induced self-assembly (PISA), surfactant-free RAFT emulsion polymerization, poly(3-vinylpyridine), amphiphilic block copolymer

## Abstract

In this work, we present a novel synthetic route to diblock copolymers based on styrene and 3-vinylpyridine monomers. Surfactant-free water-based reversible addition–fragmentation chain transfer (RAFT) emulsion polymerization of styrene in the presence of the macroRAFT agent poly(3-vinylpyridine) (P3VP) is used to synthesize diblock copolymers with molecular weights of around 60 kDa. The proposed mechanism for the poly(3-vinylpyridine)-*block*-poly(styrene) (P3VP-*b*-PS) synthesis is the polymerization-induced self-assembly (PISA) which involves the in situ formation of well-defined micellar nanoscale objects consisting of a PS core and a stabilizing P3VP macroRAFT agent corona. The presented approach shows a well-controlled RAFT polymerization, allowing for the synthesis of diblock copolymers with high monomer conversion. The obtained diblock copolymers display microphase-separated structures according to their composition.

## 1. Introduction

Free radical polymerization provides a feasible and robust route towards the polymerization of a variety of vinyl monomers. However, the inability to synthesize well-defined copolymers with a predetermined morphology turns out to be the limiting factor in this process. Especially, the lack of control over molecular weight, chain architecture and topology restrains the utility of polymers prepared by radical polymerization in many applications [1]. The necessity to govern the molecular weight, the dispersity and the molecular architecture has directed researchers towards controlled or ‘‘living’’ radical polymerization (CRP) which has developed into a versatile and widely used tool to synthesize well-defined polymer structures [2]. In addition to a variety of other controlled radical polymerization techniques, reversible addition–fragmentation chain transfer polymerization (RAFT) is of particular importance due to its functional group tolerance and the particular applicability for the synthesis of well-defined polymers [1,3,4,5,6]. Furthermore, it is the method of choice to design precisely structured block copolymers via radical polymerization [1,7]. RAFT polymerization takes place via a degenerative transfer process and relies on the use of chain-transfer (RAFT) agents, often possessing a thiocarbonylthio moiety [8]. Many research groups work on RAFT polymerization of various monomers and, especially for the case of vinyl pyridine monomers, a variety of research works can be found in the literature for the synthesis of homopolymers as well as block copolymers. [9,10,11,12]. Another relevant class of polymers is the polymers that are based on styrene. The chain growth in case of the synthesis of polystyrene has been found to be slow in radical polymerizations [13]. Aqueous RAFT emulsion polymerization, a versatile and more environmentally friendly method due to the use of water instead of organic solvent, has been proven to be a feasible approach to accelerate styrene polymerization. Emulsion polymerizations are commonly stabilized by a surfactant (e.g., low molecular sodium dodecyl sulfate) which stabilizes the emulsion at the beginning and the monomer-swollen micelles as well as the young latex particles towards the end of the polymerization [14]. However, the use of surfactants carries risks. They may separate from the particles and thus lead to particle coagulation. In addition, it is difficult to remove the surfactant from the polymer completely. The surfactant-free emulsion polymerization presents a novel approach towards the in situ synthesis of amphiphilic diblock copolymers that self-assemble into stabilized polymer particles. Generally, self-assembly describes a spontaneous process in which nanoscale units accumulate into a well-ordered arrangement to obtain minimum free energy by minimizing repulsion and maximizing attractive molecular forces, controlled by the enthalpy as well as the entropy of the system [15,16,17,18,19]. Karagoz et al. employed the innovative concept called polymerization-induced self-assembly (PISA) to synthesize polymeric nanoparticles with morphological control [20]. Polymerization-induced self-assembly is based on the chain extension of homopolymers (macroRAFT agents) with a co-monomer to yield block copolymers. According to Karagoz et al., self-assembly is induced by the insolubility of the second block in the polymerization solution during a continuous chain extension. A great practical advantage of the PISA approach is certainly the possibility of synthesizing a variety of block copolymers via a simple one-pot reaction, using only one initial homopolymer. Ferguson et al. employed a poly(acrylic acid) (PAA) macroRAFT agent to stabilize the emulsion polymerization of butyl acrylate (BA) via PISA [21]. Their emulsion system contained, besides the PAA macroRAFT agent, only the initiator and the monomer to be polymerized. Ferguson et al. could prove the self-assembly of the growing block copolymer as soon as sufficient BA units were assembled and the macroRAFT agent’s chain extension in solution was completed. Further advances in the field of PISA, such as the development of worm-like polymers, biodegradable polymers and biosynthesized molecules with application in the biomedical area, with or without the use of radical initiator have been discussed by Truong et al. [22] and Boyer et al. [23].

In this work, we describe the RAFT synthesis of diblock copolymers formed by poly(3-vinylpyridine) and polystyrene using the PISA approach. One inherent problem of surfactant-free emulsion polymerizations arises when the initial homopolymer, in our case the P3VP macroRAFT agent, is poorly water-soluble. Therefore, an organic cosolvent was used. The diblock copolymers were characterized by size-exclusion chromatography (SEC), proton nuclear magnetic resonance (^1^H-NMR) and differential scanning calorimetry (DSC). Atomic force microscopy (AFM), scanning electron microscopy (SEM) and transmission electron microscopy (TEM) provided information about their morphologies.

## 2. Materials and Methods

### 2.1. Materials

Experiments were carried out with ultrapure water (resistivity >18.2 MΩ∙cm^−1^) obtained from a Millipore (Merck, Darmstadt, Germany) Direct-Q^®^ UV water purification system. 2,2′-Azobis(2-methylpropionitrile) (AIBN) (98%, Sigma-Aldrich, Munich, Germany, stored at 4 °C), 2-cyano-2-propyl benzodithioate (CPBD) (>97%, Sigma-Aldrich, Munich, Germany, stored at 4 °C), tetrahydrofuran (THF) (99.8%, Merck) and *N,N*-dimethylformamide (DMF) (>99.5%, Merck) were used without further treatment and purification. Styrene (99%, Sigma-Aldrich, contained methyl ether hydroquinone as an inhibitor, stored at 4° C) was freshly percolated through a column of basic aluminum oxide (>98%, Sigma-Aldrich) prior to use to remove the inhibitor methyl ether hydroquinone. 1,4-Dioxane (DOX) (>99.5%, VWR Chemicals, Darmstadt, Germany) was stored over KOH pellets (>85%, Merck) and freshly percolated through a column of aluminum oxide to remove peroxides. 3-Vinylpyridine (3VP) (97%, TCI Deutschland GmbH, Eschborn, Germany) was stored and transferred under a nitrogen atmosphere. All other chemicals were used as received.

#### 2.1.1. Synthesis of the P3VP macroRAFT Agent

A typical synthesis of P3VP via RAFT polymerization was conducted as follows: 2-Cyano-2-propyl benzodithioate (CPBD) (11 mg, 49 µmol, 1.0 eq) and AIBN (8 mg, 49 µmol, 0.05 eq) were dissolved in 3VP (964 mg, 9.2 mmol, 188 eq) ((3VP)/(CPBD)/(AIBN) = 188/1/0.05). A sample for ^1^H-NMR characterization was taken prior to polymerization to serve as a reference. The solution was degassed by purging with nitrogen for 15 min at 0 °C. The bulk polymerization was conducted in a thermoshaker (Cellmedia, Elsteraue, Germany) at 80 °C for 4 h and then quenched by ice-cooling and exposure to air. Subsequently, the crude polymer was dissolved in THF and precipitated in ice-cold *n*-hexane twice. The polymer was dried in vacuum at 40 °C for 24 h and obtained as a light pink powder. Monomer conversion: 95% calculated by ^1^H-NMR. SEC: Apparent number average molecular weight: ${\bar{M}}_{n,app}$ = 18.6 kDa, (theoretical number average molecular weight (calculated by ^1^H-NMR): ${\bar{M}}_{n,th}$ = 18.9 kDa), molecular weight distribution: *Đ* = 1.1.

#### 2.1.2. Synthesis of P3VP-b-PS via RAFT Emulsion Polymerization

For a typical surfactant-free RAFT emulsion polymerization the P3VP macroRAFT agent/macro-stabilizer (113 mg, 6.1 µmol, 1.0 eq) was dissolved in DMF/H_2_O (50/50, v/v) (5 mL). AIBN (50 µg, 0.3 µmol, 0.05 eq) in DMF (100 µL) was added to the solution. Styrene (380.5 mg, 3.65 mmol, 601 eq) was added at the end. The total solids content in the formulation amounted for 10 wt%. The heterogeneous mixture was degassed by purging with nitrogen for 15 min at 0 °C and homogenized by stirring at 800 rpm using a thermoshaker. A sample for ^1^H-NMR characterization was taken prior to polymerization to serve as a reference. The subsequent polymerization was conducted for 24 h at 70 °C and 850 rpm. The polymerization was quenched by ice-cooling and exposure to air. The crude polymer was isolated by removing DMF/H_2_O under reduced pressure. The residue was dissolved in THF and the polymer was precipitated by pouring the solution into ice-cold *n*-hexane while stirring. This procedure was repeated twice. The polymer was dried in vacuum at 40 °C for 24 h and obtained as a pink-orange powder. Monomer conversion: 78%, SEC: ${\bar{M}}_{n,app}$ = 59.9 kDa (${\bar{M}}_{n,th}$ = 53.0 kDa), *Đ =* 1.1.

### 2.2. Analytics

#### 2.2.1. Nuclear Magnetic Resonance Spectroscopy (NMR)

The monomer conversion of the RAFT polymerization was determined via ^1^H-NMR spectroscopy. The NMR experiments were performed using a Bruker AV500 spectrometer (Bruker, Rheinstetten, Germany). ^1^H-NMR spectra were recorded applying a 10 ms 90° pulse at a sample temperature of 298 K. Sixteen scans were recorded with a relaxation delay of 3 s. Sample concentrations were approximately 20 g∙L^−1^ in THF-d_8_ or CDCl_3_. The conversion of styrene in the emulsion polymerization (determined in THF-d_8_) was calculated from the integral ratio of the aromatic P3VP-*b*-PS signal at 7.70–6.20 ppm (taken from the ^1^H-NMR of the diblock copolymer, Figure S2) and the aromatic P3VP signal at 7.70–6.20 ppm (displayed in the ^1^H-NMR of the P3VP macroRAFT agent precursor, Figure S1). The conversions were then estimated by comparing the integral ratios of the samples taken before and after the polymerization The conversion of 3VP was determined by ^1^H-NMR from the integral ratio of the aromatic P3VP signal at 7.60–8.45 ppm (corrected by subtraction of the monomer integrals) and the monomer signal (of one proton) at 8.55 ppm.

#### 2.2.2. Size-Exclusion Chromatography (SEC)

Size-exclusion chromatography (SEC) was conducted on a gel permeation chromatography (GPC) system, with *N,N*-dimethylacetamide (DMAc, Sigma-Aldrich, Munich, Germany) with the addition of lithium chloride. A Waters 717 Plus instrument equipped with PSS GRAM columns (GRAM pre-column (dimension 8–50 mm) and two GRAM columns of different porosity (3000 Å and 1000 Å)) with dimensions of 8 mm × 300 mm and a particle size of 10 μm was used. The flow rate was 1.0 mL/min using a VWR-Hitachi 2130 pump (VWR International, Darmstadt, Germany) and a Shodex RI-101 refractive index detector (Showa Denko Europe, Munich, Germany).

#### 2.2.3. Differential Scanning Calorimetry (DSC)

Differential scanning calorimetry was performed on a DSC 1 (Star system, Mettler-Toledo, Gießen, Germany) in the temperature range of 30 °C to 250 °C, with a heating and cooling rate of 10 K/min, using nitrogen as a purge gas stream (60 mL/min). Two heating–cooling cycles were conducted per sample. For the interpretation of the results, the second heating trace was used.

#### 2.2.4. Atomic Force Microscopy (AFM)

Atomic force microscopy was carried out on a Bruker MultiMode 8 atomic force microscope (BrukerNano, Karlsruhe, Germany) operating in PeakForce QNM^®^ mode at room temperature. SCANASYST-AIR cantilevers (spring constant 0.4 N/m, silicon tip of 2 nm radius) were used to investigate the microphase morphology of the samples. The images were evaluated by using the Nanoscope 9.2 Software (BrukerNano, Karlsruhe, Germany).

Thin films for AFM and SEM measurements were prepared as follows: Silicon (Si) wafer substrates 1 cm × 1 cm were cleaned initially with CH_2_Cl_2_, followed by cleaning with a mixture of H_2_O, H_2_O_2_ and NH_4_OH (60/20/20), and finally rinsed with distilled H_2_O and treated with H_2_/O_2_ plasma. A thin film with a thickness of approximately 100 nm was generated by spin-coating 2 wt% P3VP-*b*-PS polymer solutions in CHCl_3_ onto the Si wafers. The P3VP-*b*-PS diblock copolymers were dissolved in CHCl_3_, a rather non-selective solvent for P3VP and PS. A spin-coater G3P-8 (Specialty Coating Systems, Indianapolis, IN, USA) was operated at 3000 rpm for 60 s. The samples were stored in a small closed vessel in a dry-keeper desiccator at room temperature.

Additional thermal annealing of the samples was conducted at a temperature T_annealing_ = 150 °C under vacuum for 15 h. For microphase reconstruction, the spin-coated samples were exposed for 10 min in 1,4-dioxane vapor and dip-coated in ethanol for 5 min.

#### 2.2.5. Scanning Electron Microscopy (SEM)

A scanning electron microscope Merlin (Carl ZEISS, Oberkochen, Germany) was used to characterize the morphology of the diblock copolymer samples. Secondary electron images were taken at accelerating voltages of 0.9–3 kV of 100-nm-thick P3VP-*b*-PS films on Si wafer substrates, as prepared for the AFM measurements.

#### 2.2.6. Transmission Electron Microscopy (TEM)

The bulk morphology of the diblock copolymer was investigated via TEM employing a Tecnai G2 F20 electron microscope (Thermo Fisher Scientific, Eindhoven, the Netherlands) operated at 120 kV in bright field mode. Polymer films were cast from the CHCl_3_ solutions and slowly dried in the presence of solvent vapor in a desiccator for approximately 2 weeks. The films were further thermally annealed stepwise up to 140 °C in vacuum. Ultrathin sections of about 50 nm were cut with a Leica Ultramicrotome EM UCT (Leica Microsystems, Wetzlar, Germany) equipped with a diamond knife (Diatome AG, Biel, Switzerland). P3VP was selectively stained in I_2_-vapor for 1 h.

## 3. Results and Discussion

### 3.1. Preliminary Remarks and Solubility of Aqueous P3VP Solutions

In order to conduct the surfactant-free emulsion polymerization, the solubility of the unstabilized macroRAFT agent in water-based solution is essential at room temperature and reaction temperature. Common polymerization temperatures of RAFT polymerizations are about 65–80 °C. As already mentioned, the P3VP macroRAFT agent utilized in this work is poorly water-soluble at neutral pH value. To increase the solubility of the P3VP macroRAFT agent, a suitable organic co-solvent was used.

**Figure 1:**
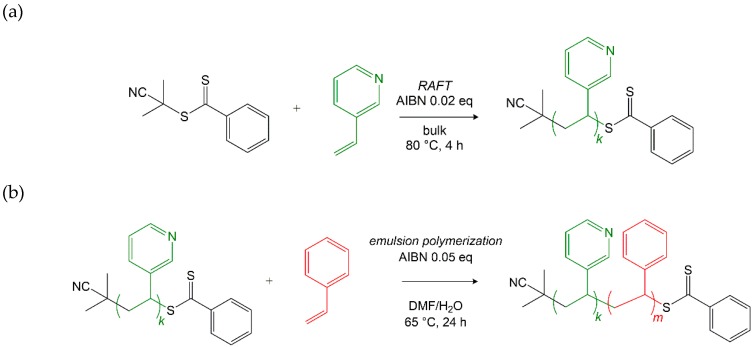
Synthetic route to the poly(3-vinylpyridine)-*block*-poly(styrene) (P3VP*-b-*PS) diblock copolymer; (**a**) synthesis of the poly(3-vinylpyridine) (P3VP) macroRAFT agent via reversible addition–fragmentation chain transfer (RAFT) bulk polymerization; (**b**) synthesis of the P3VP-*b*-PS diblock copolymer via surfactant-free RAFT emulsion polymerization.

Apart from its positive effect on the solubility of the P3VP macroRAFT agent, the cosolvent DMF led to an increase of the poor styrene solubility in the water-based reaction medium, from 3 mM in pure H_2_O to 4–5 mM in a 50/50 (v/v) DMF/H_2_O mixture [24]. The values were determined experimentally via cloud point determination. The slightly increased styrene solubility can accelerate the kinetics of the initial commonly slow chain extension of the macroRAFT agent prior to micellization, which proceeds similar to a RAFT solution polymerization and is hence faster with increased effective monomer concentration. However, the styrene solubility in the chosen reaction medium is kept sufficiently low to justify a true emulsion polymerization mechanism [14,25]. Figure 1 presents the synthetic route to the pursued P3VP-*b*-PS diblock copolymer. The first step, the synthesis of the P3VP macroRAFT agent via RAFT polymerization in bulk, is followed by the surfactant-free RAFT emulsion polymerization of styrene in the presence of the P3VP macroRAFT agent/macro-stabilizer.

### 3.2. Mechanism of RAFT Emulsion Polymerization

Prior to emulsion polymerization, 3-vinylpyridine (3VP) was polymerized in bulk via RAFT to a conversion of 95%, resulting in the P3VP macroRAFT agent. 2-Cyano-2-propyl benzodithioate (CPBD) was used as RAFT agent and AIBN was added in low amounts as the initiator of the reaction ((3VP)/(CPBD)/(AIBN) = 188/1/0.05). ${\bar{M}}_{n,app}$ = 18.6 kDa (${\bar{M}}_{n,th}$ = 18.9 kDa), *Đ* = 1.1.

**Figure 2:**
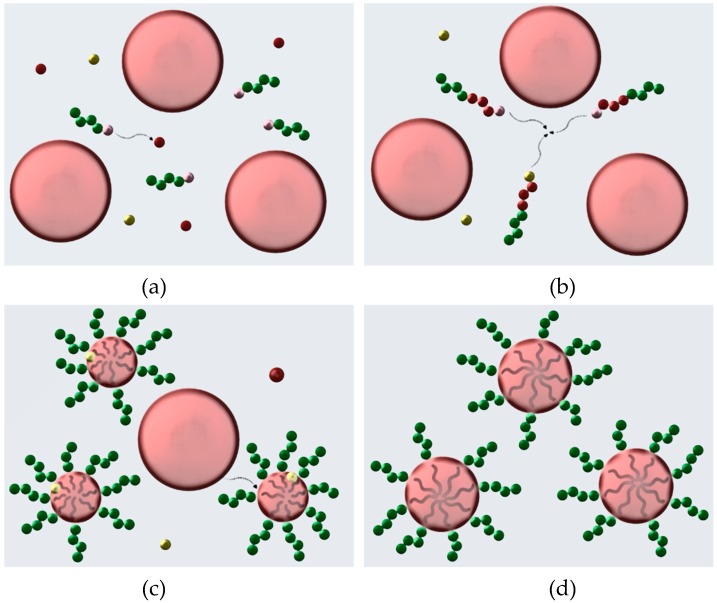
Illustration of the polymerization mechanism to the targeted diblock copolymer P3VP-*b*-PS via surfactant-free RAFT emulsion polymerization. The colors used are partially a continuity of the ones used for the reactants in the reaction schemes (Figure 1). AIBN is depicted as yellow spheres and the P3VP macroRAFT agent as a chain of green spheres with pink RAFT agent end groups. Large red spheres represent styrene droplets while small red spheres display single styrene monomer units. The grey background presents the *N,N*-dimethylformamide (DMF) /H_2_O mixture. Initially, the P3VP macroRAFT agent is dissolved in a DMF/H_2_O (50/50, v/v) solution. The addition of styrene to the P3VP macroRAFT agent leads to a macroscopic phase separation without mechanical agitation (stage 0). (**a**) The polymerization starts in solution (stage 1) until (**b**) a transition to micellization occurs. (**c**) Stage 2 is characterized by monomer migration and polymerization in micelles, until (**d**) all styrene monomer droplets are depleted, and particle formation is completed. The final latex contains PS-core particles with a P3VP macroRAFT agent corona.

For the RAFT emulsion polymerization, the P3VP macroRAFT agent/macro-stabilizer was dissolved in a DMF/H_2_O (50/50, v/v) solution. AIBN and styrene were added to the solution. The total solids content (initiator and macroRAFT agent) in the formulation amounted for 10 wt%. The surfactant-free aqueous RAFT emulsion polymerization was conducted at 65 °C for 24 h, using a thermoshaker for homogeneous mixing. In Figure 2a–d, the different stages of the polymerization mechanism are depicted. Figure 2a, which corresponds to the first mechanistic stage, implies the chain extension of the polystyrene block, which remains soluble up to a certain block length.

During chain extension of the P3VP macroRAFT agents with a few styrene monomer units, the effective styrene concentration in the reaction medium is at a constant low level. Hence, the chain transfer from oligomeric PS radicals to dormant P3VP chains is more likely and thus preferred over PS chain propagation [14,26,27]. Figure 2b shows the transition from the first to the second mechanistic stage, determined by the self-assembly of the growing diblock copolymers into micelles. Figure 2c shows the second mechanistic stage, which is characterized by the migration of styrene molecules into the micelles and, simultaneously, growth of the diblock copolymers. In the micelles, the monomer that is being converted into the polymer is continuously restocked with entering monomer droplets, keeping the monomer concentration in the growing diblock copolymer micelles constant. In the third mechanistic stage (Figure 2d) all monomer droplets are consumed, and the particle formation is completed because all micelles contain sufficient hydrophobic components to become immobile. An accompanying change in the reaction kinetics from mechanistic stage one to three can be explained as follows: As soon as sufficient hydrophobic units are assembled by macroRAFT agent chain extension and a critical block length is exceeded, self-assembly of the growing diblock copolymers occurs. The polymerization continues inside the diblock copolymer micelles, faster by a few orders of magnitude [14].

A not inconsiderable contribution to enhanced micelle stability is provided by the macroRAFT agent end groups, which are located at the interface between the particle and the aqueous phase. Despite making up only a small volume fraction of the polymer, the terminal groups significantly affect the conformation behavior and self-assembly of macroRAFT agents [28,29]. Akpinar et al. and Thompson et al. have exploited RAFT aqueous emulsion polymerization using 2-cyano-2-propyl dithiobenzoate as initial RAFT agent to prepare almost monodisperse sterically stabilized spherical diblock copolymer nanoparticles [30,31].

Styrene is preferably chosen as the monomer because of its strong hydrophobic nature [32]. In emulsion polymerizations, a distinction is made depending on the size of the ‘latex’ particles. A mini-emulsion polymerization defines the formation of surfactant- or macroRAFT agent-stabilized monomer droplets of size ranging between 50 to 500 nm whereas the ‘latex’ particles formed in a microemulsion polymerization are in the range of 20 to 40 nm [33]. Micro- or mini-emulsion polymerizations take place within the monomer-swollen micelles and are probably the most commonly used techniques in combination with RAFT polymerization [32,34].

The chosen initiator AIBN is added in very low amounts to the reaction mixture ((Styrene)/(AIBN) = 601/0.05). AIBN is poorly water-soluble but fully soluble in organic solvents like DMF. It is thus completely dissolved in the DMF/water mixture, as DMF is fully miscible with water. Upon adding styrene to the reaction mixture, AIBN is distributed between styrene and the DMF/water phase. Our investigations concerning AIBN show that an organic solvent soluble initiator, such as AIBN, which is dissolved only slightly in water is suitable for RAFT emulsion polymerizations. Other groups found the same results for ATRP and free radical emulsion polymerizations [35,36].

### 3.3. Synthesis Results of the P3VP-b-PS Diblock Copolymers

**Figure 3:**
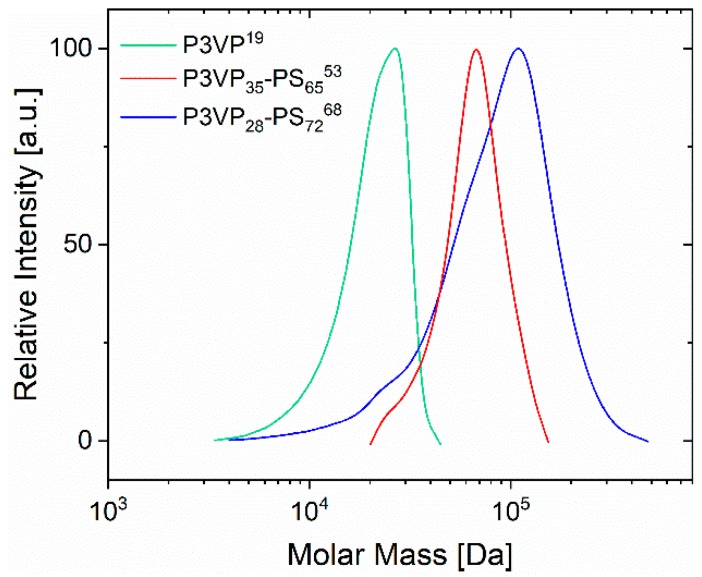
The GPC chromatograms of the obtained P3VP^19^ homopolymer (green color), the precursor of the two diblock copolymers, the P3VP_35_-PS_65_^53^ diblock copolymer (red color) and the P3VP_28_-PS_72_^68^ (blue color). The shift of the diblock copolymer curves towards higher molar mass indicates the successful synthesis upon completion of the RAFT emulsion polymerization, however, with little low-molecular-weight tailing, due to the presence of unreacted P3VP homopolymer. A good extent of control in the emulsion polymerization system is indicated by reasonable dispersities (*Đ* = 1.1 and *Đ =* 1.5).

As discussed before, P3VP acted as both macroRAFT agent and macro-stabilizer in the RAFT emulsion polymerization of styrene. The conversion of styrene polymerization in H_2_O with DMF as co-solvent was 75% for the sample P3VP_35_-PS_65_^53^ and 78% for the sample P3VP_28_-PS_72_^68^. The apparent number-average molecular weights (${\bar{M}}_{n,app}$) of P3VP-*b*-PS were obtained by GPC, calibrated with PS standards (Table 1). The molecular weight dispersities (*Đ*) are moderate with *Đ* = 1.1–1.5. The PS weight fraction in the synthesized diblock copolymers amounts for approximately 65–70 wt%, as obtained by ^1^H-NMR (Figure S2). The GPC chromatograms of the obtained P3VP homopolymer and the P3VP_35_-PS_65_^53^ and P3VP_28_-PS_72_^68^ diblock copolymers reveal that the curve corresponding to the diblock copolymer has significantly shifted towards higher molecular weights with little low-molecular-weight tailing, which indicates good control of the P3VP chain extension (Figure 3). The P3VP_28_-PS_72_^68^ diblock copolymer with the higher PS content exhibits a higher molecular weight dispersity. It can probably be attributed to a slight impurity which led to absolute control of the molecular weight distribution not being maintained regardless of the fact that the molecular weight attains the planned molecular weight value for the synthesis.

### 3.4. Thermal and Morphological Characterization of P3VP-b-PS Diblock Copolymers

Due to the characteristics of the two blocks and the knowledge that is existing on similar systems such as PS-*b*-P2VP and PS-*b*-P4VP [37,38,39] it is expected that this type of diblock copolymer under certain criteria (e.g., molecular weight) will also exhibit microphase separation and will self-assemble to a variety of structures depending on composition [40]. The microphase separation was studied experimentally via DSC, AFM, SEM and TEM.

#### 3.4.1. Thermal Analysis

**Figure 4:**
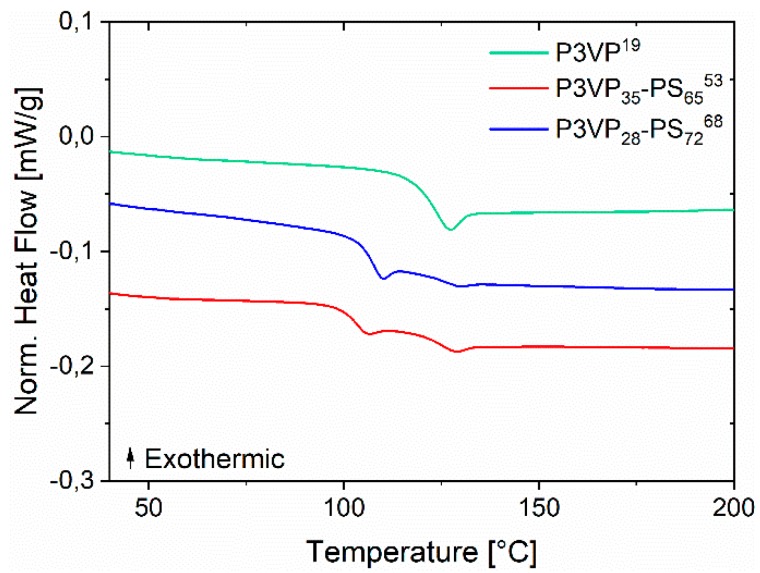
DSC thermogram of the P3VP-*b*-PS diblock copolymers and the corresponding homopolymer precursor. The two different glass transition temperatures of the two blocks are observed. The heating rate was 10 K/min.

The thermal behavior of the P3VP-*b*-PS diblock copolymers was examined by DSC experiments in the range of 30 to 200 °C. As displayed in Figure 4 and Table 2, two glass transition temperatures corresponding to the two different blocks were detected. Due to the large fraction of the PS block in the diblock copolymer, the change in the heat flow around the glass transition temperature of PS (temperature range 102–106 °C) is significantly larger than that of the P3VP. A temperature difference of almost 20 °C for the P3VP-*b*-PS diblock copolymer between the two $T_{g}s$ indicates a microphase separation between the two blocks.

#### 3.4.2. Morphological Characterization

The occurrence of microphase separation of P3VP-*b*-PS was studied in thin films and bulk.

**Figure 5:**
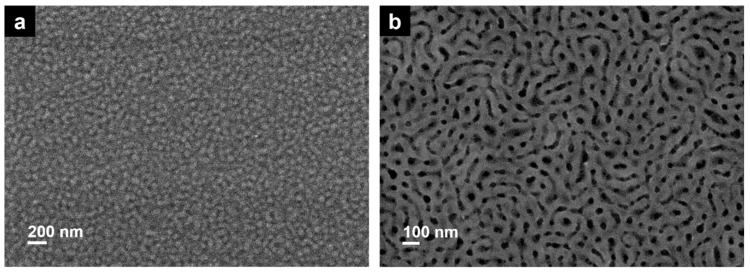
SEM micrographs of a spin-coated thin film of a 2wt % P3VP_35_-PS_65_^53^ solution (**a**) before and (**b**) after thermal annealing at *T*_annealing_ = 150 °C (15 h) combined with 10 min 1,4-dioxane vapor annealing and 5 min dip-coating in ethanol.

After thermal analysis, theoretical considerations concerning the Flory–Huggins parameters of P3VP-*b*-PS diblock copolymers represent an indication of the separation behavior of the diblock copolymers synthesized via RAFT emulsion polymerization. While the Flory–Huggins parameter between PS and P4VP is on the order of $\chi_{S,4VP}=0.35$ (at 160 °C), the interaction parameter for polystyrene and poly(2-vinylpyridine) is only about $\chi_{S,2VP}=0.1$ (at 178 °C) [37,38,39]. To the best of our knowledge, so far, no Flory–Huggins interaction parameter has been determined for a PS and P3VP system. We assume that the value of the Flory–Huggins interaction parameter $\chi_{S,3VP}$ should be found between 0.1 ($\chi_{S,2VP}$) and 0.35 ($\chi_{S,4VP}$). Scanning electron microscopy (SEM) and atomic force microscopy (AFM) were employed to study thin films. As mentioned before, CHCl_3_ was used because it is a rather non-selective solvent for both the P3VP and the PS block. Due to its low boiling point, it provides good thin-film formation. Both samples were examined as thin films. The sample P3VP_35_-PS_65_^53^ that exhibits a low molecular weight dispersity value provided better thin-film formation properties and is presented below. The SEM micrograph of a P3VP_35_-PS_65_^53^ film (Figure 5a) shows a distinct cylindrical microphase separation for the thin diblock copolymer film without long-range order. The absence of long-range order is probably caused by the preparation method, which does not allow for equilibration, due to immediate solvent evaporation after spin-coating.

Thermal or solvent vapor annealing are approaches to influence or equilibrate the morphology of a thin block copolymer film and thus to adopt a better-organized structure with fewer defects. Often, diblock copolymer morphologies in a thermodynamic equilibrium state are established by a thermal annealing process conducted in vacuum above the glass transition temperature (*T*_g_) of both blocks [41]. *T*_annealing_ must be chosen between *T*_g_ and the disordering transition temperature. Sometimes, however, the thermal annealing process does not result in a long-range order due to kinetic hindrance [41]. Thus, the equilibrium state may not be established and structural defects will be present [17]. If the system above *T*_g_ is close to the order–disorder transition the thermodynamic driving force for the improvement of the microphase order may be also reduced, which also may be a result when the film is subjected to solvent vapor annealing due to a reduced effective χ-parameter.

**Figure 6:**
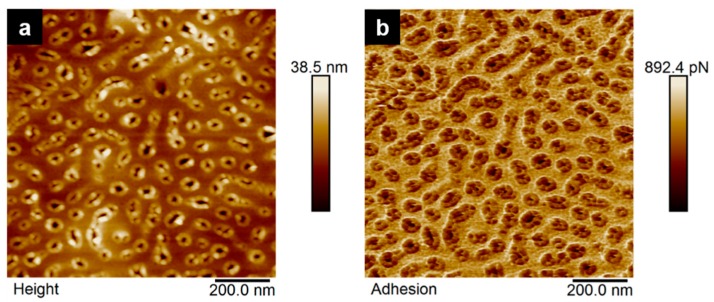
A spin-coated thin film of a 2 wt% P3VP_35_-PS_65_^53^ solution after thermal annealing at *T*_annealing_ = 150 °C (15 h) combined with 10 min 1,4-dioxane vapor annealing and 5 min dip-coating in ethanol. (**a**) Surface topography via QNM AFM height images (1 µm × 1 µm); (**b**) QNM AFM adhesion images (1 µm × 1 µm) of P3VP_35_-PS_65_^53^.

After spin-coating, the sample was annealed above the glass transition temperature $T_{g}$ of all blocks at *T*_annealing_ = 150 °C under vacuum for 15 h. An adequate long annealing time should accelerate the chain mobility. In order to further influence the diblock copolymer morphology, thermal annealing was combined with solvent vapor annealing. This treatment has been developed into a common tool to induce long-range order of diblock copolymer nanostructures [41,42,43,44,45]. Solvent annealing imparts mobility of the different polymer blocks, allowing them to attain equilibrium morphology within hours or days. The solvent vapors interact with the polymer film and often lead to a long-range order and an elimination of structure defects. Microscopically, the individual microdomains of the diblock copolymer are swollen and dried during the process. Solvent annealing is versatile since it can be used with miscellaneous diblock copolymers without elaborate pretreatment [41]. The result of the thermal annealing is presented in Figure S3 of the Supplementary Materials. It is shown that the thermal annealing led to a smooth surface without the indication of microphase separation. Thermal annealing at *T*_annealing_ = 150 °C for 15 h was then followed by 10 min of 1,4-dioxane (a selective solvent for PS) vapor annealing (in order to influence the mobility of the PS matrix and to trigger microphase separation of the block. The PS phase was softened in the presence of 1,4-dioxane vapor, leading to chain orientation mobility of the P3VP domains in the PS matrix, which is swollen and softened. The result of the annealing with 1,4-dioxane is presented in Figure S4 indicating as well that no long-range order or clear microphase separation structure can be observed, nevertheless, the surface becomes less smooth due to the swelling of the matrix. Finally, the films were dip-coated for 5 min in ethanol, a selective solvent for P3VP. Ethanol softens and swells the P3VP blocks leading to a descent of the P3VP phase and thus to a pronounced surface topography. The process of surface reconstruction by solvent annealing was formerly exploited by other groups [46,47]. Park et al. have shown that utilizing a selective solvent for thin films of a PS-*b*-P4VP diblock copolymer did not modify the order or orientation of the microdomains [47]. When the solvent annealed films were dip-coated in ethanol, a good solvent for P4VP and a non-solvent for PS, a surface reconstruction of the films could be observed displaying a distinct microstructure. Figure 5b shows an SEM micrograph of a spin-coated thin film of P3VP_35_-PS_65_^53^ after thermal annealing at *T*_annealing_ = 150 °C combined with 10 min 1,4-dioxane vapor annealing and 5 min dip-coating in ethanol. The implementation of the surface reconstruction process on P3VP_35_-PS_65_^53^ led to the formation of a film with sections of disordered hexagonally arranged cylinders. In the AFM height and adhesion images (Figure 6a,b, respectively) the hexagonally packed P3VP cylinders in the PS matrix are displayed at higher magnification. The detailed view verifies the optimization of the order of the structure, nevertheless, it indicates that it was not possible to obtain within this time the optimum structure. Extended solvent annealing time results in dewetting of the polymer films from the silicon wafer and makes the morphological characterization impossible.

**Figure 7:**
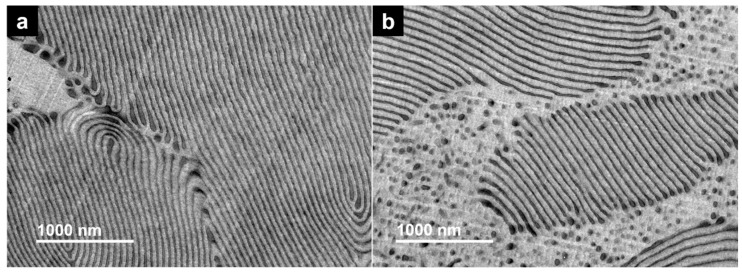
Transmission electron micrographs display the microphase separation of P3VP-*b*-PS diblock copolymer annealed at 140 °C; (**a**) P3VP_35_-PS_65_^53^; (**b**) P3VP_28_-PS_72_^68^. The bright areas correspond to the polystyrene microphase.

In order to study the bulk morphology, TEM measurements were performed on block copolymer films obtained from solution casting using a rather non-selective solvent, which was slowly evaporated in the presence of solvent vapor and further annealed above the glass transition temperature of both blocks. Presumably, the morphologies observed in the TEM micrographs (Figure 7) display the equilibrium morphologies of the diblock copolymers, even if they are not free of morphological defects. For a P3VP weight fraction of 35% and an apparent molecular weight of 53 kDa (P3VP_35_-PS_65_^53^), the lamellar structure was found. Concerning the second sample with an apparent molecular weight of 68 kDa and a P3VP weight fraction of 28% (P3VP_28_-PS_72_^68^) the morphology identified is the cylindrical, which is depicted in the TEM figure with large areas identified as cylindrical domains of P3VP cylinders in a continuous PS matrix oriented parallel to the plane, while a domain of dark dots indicates the perpendicular oriented P3VP cylinders in the PS matrix. The grey areas in Figure 7a can be attributed to short-chain P3VP-*b*-PS portions, with high PS content, (see low-molecular-weight tailing in GPC measurements, Figure 3) which are possibly beyond the order–disorder transition and thus no microphase separation can occur. The found morphologies agree well with the typically expected structures for microphase-separated diblock copolymers with these compositions [48].

## 4. Conclusions

In this work, P3VP-*b*-PS diblock copolymers were synthesized via water-based surfactant-free RAFT emulsion polymerization. The novel and more environmentally friendly emulsion polymerization of styrene in the presence of a P3VP macroRAFT agent generated diblock copolymers with molecular weights of around 60 kDa within one day. For successful emulsion polymerization, the addition of an organic cosolvent (DMF) was necessary to solubilize the medium molecular weight P3VP macroRAFT agent. The proposed mechanism for the P3VP-*b*-PS synthesis is the polymerization-induced self-assembly (PISA) which involves the in situ formation of micellar objects through initial chain growth in the aqueous phase. This formation is followed by self-assembly of the growing diblock copolymers, that consist of a PS core and a stabilizing P3VP macroRAFT agent corona.

The P3VP-*b*-PS diblock copolymers show microphase separation due to the incompatibility of the covalently linked P3VP and PS blocks, similar to P2VP-*b*-PS and P4VP-*b*-PS diblock copolymers. The morphology was studied both in bulk and in thin films. While the slow solvent evaporation led to rather well-ordered structures in the bulk state, the spin-coating of thin films led to poorly ordered morphologies. Subsequent thermal and solvent vapor annealing improved the order in the thin films and resulted in similar morphologies as obtained from the bulk state.

## Figures and Tables

**Table 1:** GPC results for the synthesized P3VP-*b*-PS diblock copolymers.

Polymer ^a^	${\bar{M}}_{n,app}$^b^ (kDa)	${\bar{M}}_{w,app}$^b^ (kDa)	${\bar{M}}_{n,th}$^c^ (kDa)	*Đ*
P3VP_35_-PS_65_^53^	60	68	53	1.1
P3VP_28_-PS_72_^68^	64	99	68	1.5

^a^ P3VP_x_PS_y_^z^: x: wt% P3VP, y: wt% PS, z: ${\bar{M}}_{n,\mathrm{th}}$. ^b^ The apparent molecular weights ${\bar{M}}_{n,\mathrm{app}}$ and ${\bar{M}}_{w,\mathrm{app}}$ were determined by GPC (eluent: DMAc) calibrated with PS standards. ^c^ ${\bar{M}}_{n,\mathrm{th}}$ was calculated as follows: ${\bar{M}}_{n,\mathrm{th}}=\frac{\left[monomer\right]}{\left[RAFT\right]}\times M_{monomer}\times monomer\;conversion+M_{RAFT}$.

**Table 2:** The experimental glass transition temperatures ($T_{g}$ of P3VP-*b*-PS and the related homopolymer obtained from DSC are shown.

Polymer	${\bar{M}}_{n,th}$ (kDa)	*T_g_*_,PS_ (°C)	*T_g_*_,P3VP_ (°C)
P3VP^19^	19	NA	121
P3VP_35_-PS_65_^53^	53	102	122
P3VP_28_-PS_72_^68^	68	106	123

## Supplementary Materials

The following are available online at https://www.mdpi.com/1996-1944/12/19/3145/s1, Figure S1. ^1^H-NMR spectra of crude (top, in THF-d_8_) and precipitated (bottom, in CDCl_3_) poly(3-vinylpyridine) (P3VP) macroRAFT agent. The conversion of 3VP was determined by ^1^H-NMR from the integral ratio of the aromatic P3VP signal at 7.60–8.45 ppm (corrected by subtraction of the monomer integrals) and the monomer signal (of one proton) at 8.55 ppm; Figure S2. Exemplary ^1^H-NMR spectra in THF-d_8_ respectively CDCl_3_ of a crude (top) and precipitated (bottom) poly(3-vinylpyridine)-*b*-polystyrene (P3VP*-b-*PS) diblock copolymer after polymerization via surfactant-free RAFT emulsion polymerization. The conversion of styrene in the emulsion polymerization was calculated from the integral ratio of the aromatic P3VP-*b*-PS signal at 7.70–6.20 ppm (taken from the ^1^H-NMR spectrum of the diblock copolymer) and the aromatic P3VP signal at 7.70–6.20 ppm (displayed in the ^1^H-NMR spectrum of the P3VP macroRAFT agent precursor); Figure S3. A spin-coated thin film of a 2 wt% P3VP_35_-PS_65_^53^ solution after thermal annealing at *T*_annealing_ = 150 °C (15 h). (**a**) Surface topography via QNM AFM height images (1 µm × 1 µm); (**b**) QNM AFM adhesion images (1 µm × 1 µm) of P3VP_35_-PS_65_^53^; Figure S4. A spin-coated thin film of P3VP_35_-PS_65_^53^ after thermal annealing at *T*_annealing_ = 150 °C (15 h) combined with 10 min 1,4-dioxane vapor annealing. (**a**) Surface topography via QNM AFM height images (1 µm × 1 µm); (**b**) QNM AFM adhesion images (1 µm × 1 µm) of P3VP_35_-PS_65_^53^.
