# Supplementary material for: Synthesis of Poly(3-vinylpyridine)-Block-Polystyrene Diblock Copolymers via Surfactant-Free RAFT Emulsion Polymerization
Source: Materials (Basel). 2019 Sep 26;12(19):3145. doi: 10.3390/ma12193145 (PMC6803976; doi:10.3390/ma12193145)
Supplement: Supplementary file 1 [file materials-12-03145-s001.pdf]

materials

Supplementary Materials

Synthesis of Poly(3-vinylpyridine)-*block*-polystyrene Diblock Copolymers via Surfactant-Free RAFT Emulsion Polymerization

Katharina Nieswandt [1], Prokopios Georgopanos [1,*], Clarissa Abetz [1], Volkan Filiz [1] and Volker Abetz [1,2,*]

1 Helmholtz-Zentrum Geesthacht, Institute of Polymer Research, Max-Planck-Straße 1, 21502 Geesthacht, Germany
2 Institute of Physical Chemistry, University of Hamburg, Martin-Luther-King-Platz 6, 20146 Hamburg, Germany
* Correspondence: prokopios.georgopanos@hzg.de (P.G.), volker.abetz@hzg.de (V.A.)
Tel.: +49-4152-87-2420 (P.G.) +49-4152-87-2461 (V.A.) Fax: +49-4152-87-2499

NMR spectra

^{1}H-NMR spectra of poly(3-vinylpyridine) and poly(3-vinylpyridine)-*b*-polystyrene (P3VP-*b*-PS) diblock copolymer.

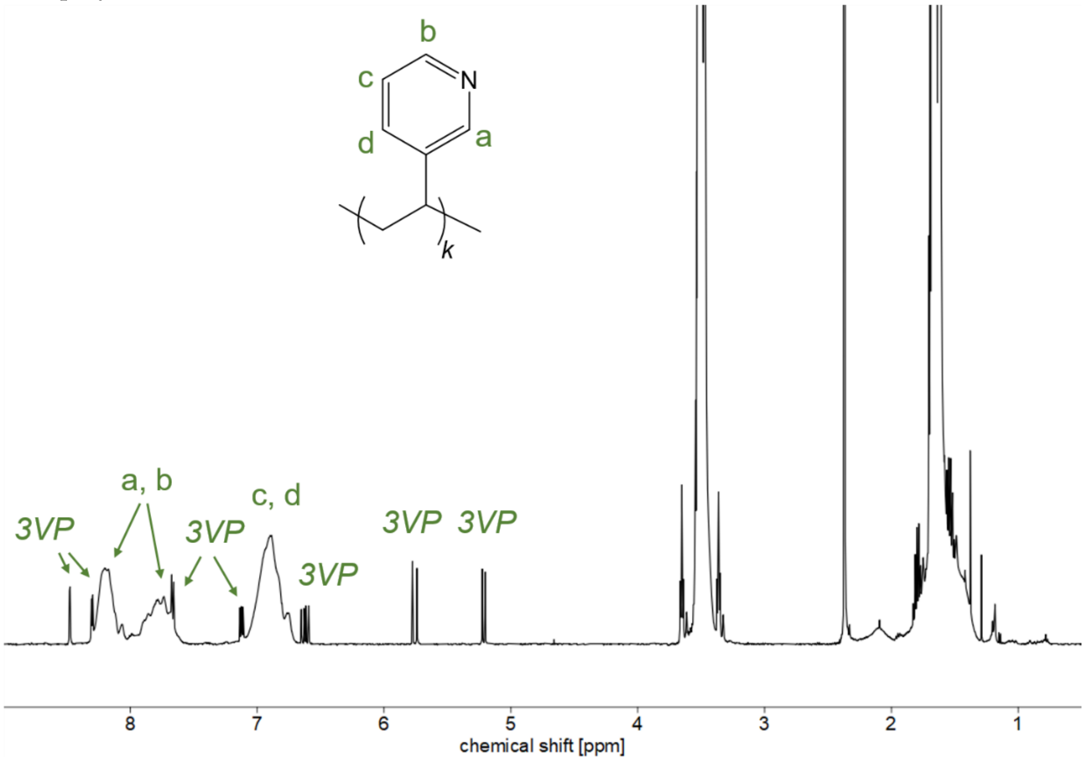

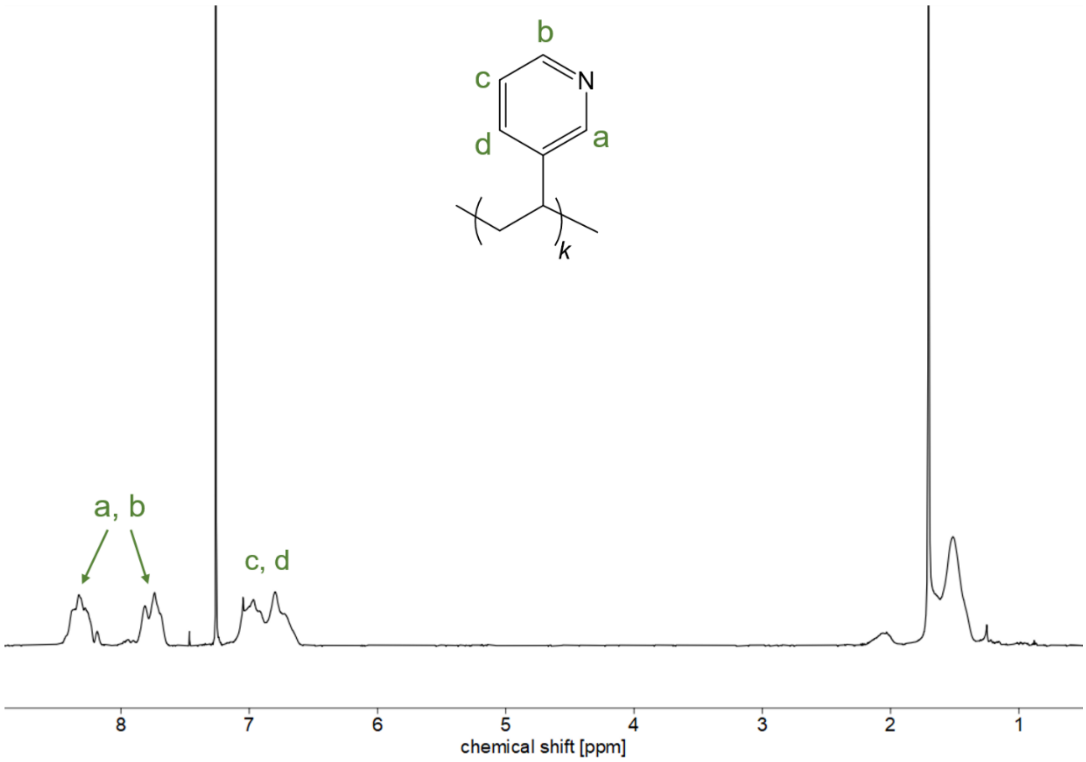

Figure S1. ^{1}H-NMR spectra of crude (top, in THF-d_8) and precipitated (bottom, in $CDCl_3$) poly(3-vinylpyridine) (P3VP) macroRAFT agent. The conversion of 3VP was determined by ^{1}H-NMR from the integral ratio of the aromatic P3VP signal at 7.60–8.45 ppm (corrected by subtraction of the monomer integrals) and the monomer signal (of one proton) at 8.55 ppm.

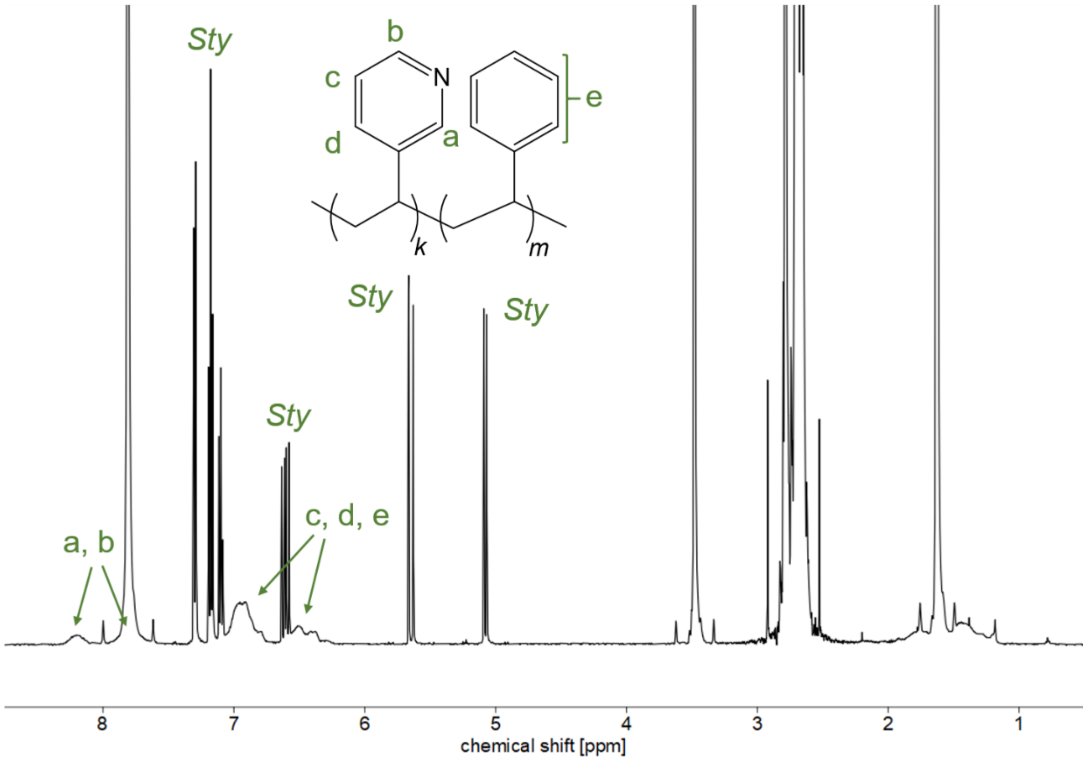

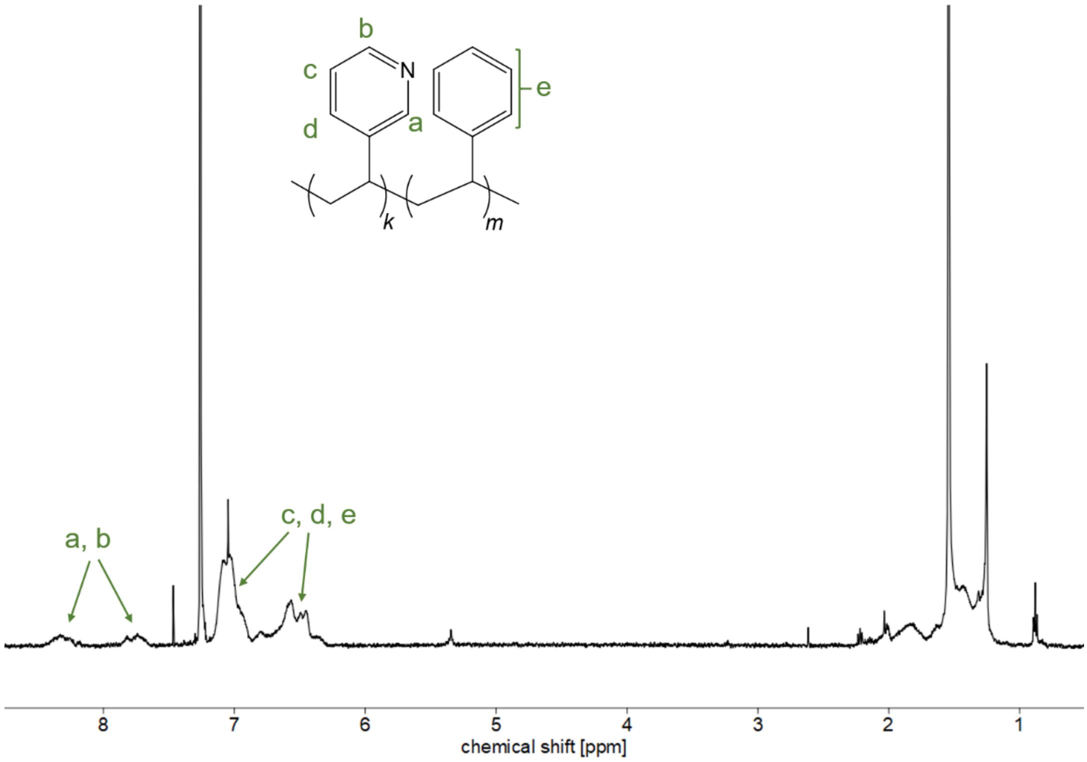

Figure S2. Exemplary ^{1}H-NMR spectra in THF-d_8 respectively $CDCl_3$ of a crude (top) and precipitated (bottom) poly(3-vinylpyridine)-*b*-polystyrene (P3VP-*b*-PS) diblock copolymer after polymerization via surfactant-free RAFT emulsion polymerization. The conversion of styrene in the emulsion polymerization was calculated from the integral ratio of the aromatic P3VP-*b*-PS signal at 7.70–6.20 ppm (taken from the ^{1}H-NMR spectrum of the diblock copolymer) and the aromatic P3VP signal at 7.70–6.20 ppm (displayed in the ^{1}H-NMR spectrum of the P3VP macroRAFT agent precursor).

Morphological Characterization

Additional characterization of the intermediate annealing steps was performed via AFM. In the AFM height and adhesion images below, the results from the individual annealing steps (thermal annealing at $T_{annealing}$ = 150 °C for 15 h and thermal annealing followed by 1,4-dioxane vapor annealing for 10 min) are presented. No pronounced microphase separation or long-range order was found after thermal annealing (Figure S1). The same result was observed after thermal annealing combined with 1,4-dioxane vapor annealing (Figure S2).

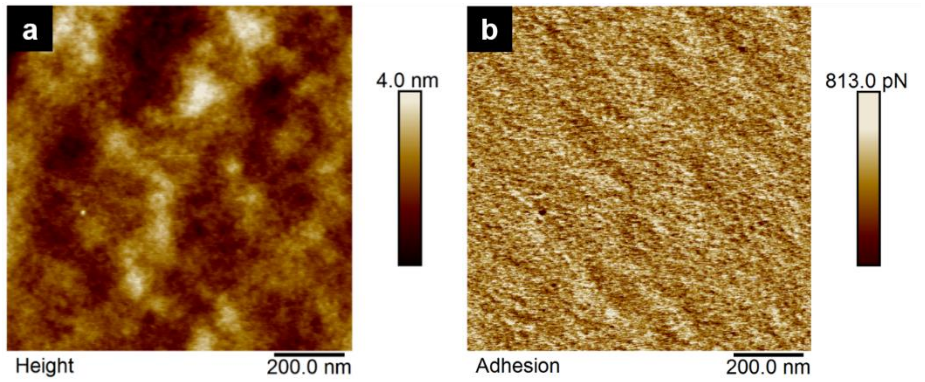

Figure S3. A spin-coated thin film of a 2 wt% $P3VP_{35}$-PS_{65}^{53} solution after thermal annealing at $T_{annealing}$ = 150 °C (15 h). (**a**) Surface topography via QNM AFM height images (1 µm × 1 µm); (**b**) QNM AFM adhesion images (1 µm × 1 µm) of $P3VP_{35}$-PS_{65}^{53}.

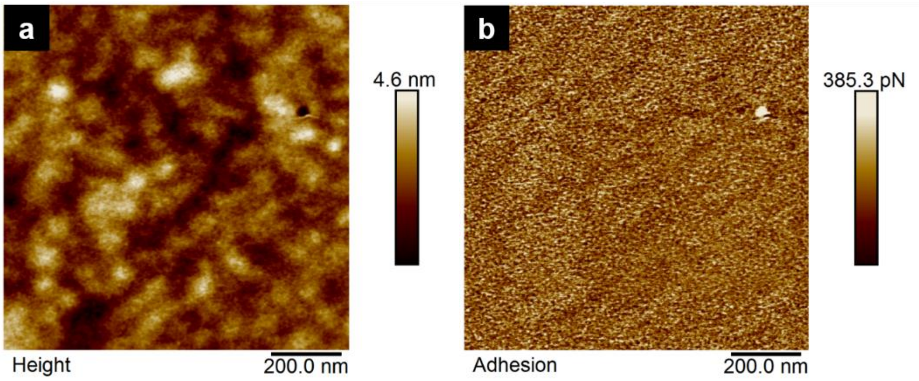

Figure S4. A spin-coated thin film of $P3VP_{35}$-PS_{65}^{53} after thermal annealing at $T_{annealing}$ = 150 °C (15 h) combined with 10 min 1,4-dioxane vapor annealing. (**a**) Surface topography via QNM AFM height images (1 µm × 1 µm); (**b**) QNM AFM adhesion images (1 µm × 1 µm) of $P3VP_{35}$-PS_{65}^{53}.
